# Process Parameter Prediction for Fused Deposition Modeling Using Invertible Neural Networks

**DOI:** 10.3390/polym15081884

**Published:** 2023-04-14

**Authors:** Lukas Pelzer, Andrés Felipe Posada-Moreno, Kai Müller, Christoph Greb, Christian Hopmann

**Affiliations:** 1 Institute for Plastics Processing, RWTH Aachen University, 52074 Aachen, Germany; 2 Institute for Data Science in Mechanical Engineering, RWTH Aachen University, 52068 Aachen, Germany; andres.posada@dsme.rwth-aachen.de; 3 Institut für Textiltechnik, RWTH Aachen University, 52074 Aachen, Germany; kai.mueller@ita.rwth-aachen.de (K.M.);

**Keywords:** additive manufacturing, fused deposition modeling, neural network, part quality, process parameters, production management

## Abstract

Additive manufacturing has revolutionized prototyping and small-scale production in the past years. By creating parts layer by layer, a tool-less production technology is established, which allows for rapid adaption of the manufacturing process and customization of the product. However, the geometric freedom of the technologies comes with a large number of process parameters, especially in Fused Deposition Modeling (FDM), all of which influence the resulting part’s properties. Since those parameters show interdependencies and non-linearities, choosing a suitable set to create the desired part properties is not trivial. This study demonstrates the use of Invertible Neural Networks (INN) for generating process parameters objectively. By specifying the desired part in the categories of mechanical properties, optical properties and manufacturing time, the demonstrated INN generates process parameters capable of closely replicating the desired part. Validation trials prove the precision of the solution with measured properties achieving the desired properties to up to 99.96% and a mean accuracy of 85.34%.

## 1. Introduction

Additive Manufacturing (AM) is a layer-based, tool-less production technology. This means that arbitrary, three-dimensional geometries can be manufactured on the same machine without the need for manual set-up processes or manufacturing tools and molds. With this technology, it is possible to produce complex shapes and to create internal geometries inside objects which could otherwise not be manufactured. Historically, AM is often used for prototyping; however, technological advances are making the process suitable for larger production runs of end-use parts [1,2]. Especially Fused Deposition Modeling (FDM), characterized by an extrusion unit depositing a molten, thermoplastic material and joining it layer-by-layer, offers a lot of flexibility and freedom, making it the most used technology in its domain [3].

The flexibility becomes apparent in the number of process parameters which need to be set prior to production. Those parameters depend on the machine and the material being used, as well as the desired part properties. They influence part strength, surface roughness and manufacturing time, amongst other things [4,5,6,7]. Their influence on the properties of the created parts is significant [7]. For example, a study varying raster angles have shown a nearly 100% increase in tensile strength between the worst and best-achieved result [8]. By varying build direction, infill percentage, manufacturing speed, extrusion temperature, layer height and infill pattern, Alafaghani et al. were able to achieve Young’s moduli starting from 1947.05 MPa up to 3177.53 MPa [5]. Regarding the build orientation of the part, a 45.8% drop in tensile strength can be noted between the best and worst orientation [9], while manufacturing time is also greatly affected [10]. Furthermore, process parameters have interdependencies regarding the created part properties. For example, increasing process speed typically decreases manufacturing time but at the same time reduces mechanical properties [6,11], while increasing nozzle temperature typically increases mechanical properties [6]. This means that, in theory, a higher temperature can be used to mitigate at least one downside of using a higher manufacturing speed. However, the actual temperature increase has to be determined for each machine and for each material. This also complicates setting process parameters systematically since they cannot be set and locked in one by one because of interdependencies. Lastly, there is no single best set of parameters for FDM. The best possible set of parameters always depends on the desired outcome. For example, there is a conflict of goals regarding good mechanical (e.g., tensile strength, Young’s modulus) and optical properties (e.g., dimensional accuracy, surface roughness) [12]. By choosing a high-temperature level for the material extrusion, good mechanical properties can be achieved because higher temperatures support the interdiffusion of the polymer chains. However, this measure requires more time for the solidification of the material, leading to a longer formable, uncontrollable state, degrading optical properties and dimensional accuracy. Choosing a low-temperature level, on the other hand, has the inverse effect. This way, the material solidifies quickly in its intended position, allowing for a good surface finish and dimensional accuracy, but leaving little time for adequate layer bonding, therefore reducing mechanical properties [12]. This example shows the difficulty and the need for technological expert knowledge when setting up the process. A single best manufacturing profile does not exist. Instead, adjusting the set of parameters with regard to the desired part properties is necessary.

**Figure 1:**
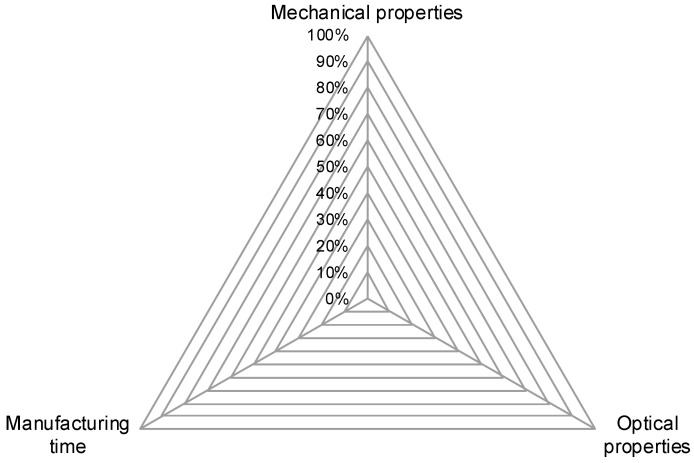
Triangle of goals for FDM, depicted as radial chart. Not all properties can be reached optimally at the same time.

This leads to three superordinate goals which are in conflict with each other. The triangle shown in Figure 1 visualizes these goals regarding part properties in FDM. The three corner points are good mechanical properties, good optical properties and short manufacturing time. Because of the interdependencies of process parameters and the opposing effects those parameters can have on part properties, this triangle of goals cannot be filled completely. For each part being produced, a trade-off has to be made. From a productivity point of view, this means that manufacturing time should always be as low as possible while retaining the minimum needed mechanical and optical properties.

Up until now, the selection of process parameters for FDM was based on expert knowledge [13]. Experienced users with a good knowledge of the process are typically able to achieve adequate to good results from low-cost or even mechanically bad machines, while inexperienced users can obtain bad to useless results from even the most expensive machines. This problem is increased by the fact that traditional methods to calculate and design parts based on their intended use- and load-case cannot be applied to FDM since the material is not homogeneously distributed in part [14]. This means that testing material properties using a solid part and then extrapolating those values with regard to the final geometry does not work. Furthermore, the complex phenomenon of layer bonding cannot be modeled sufficiently to allow for simulation software to aid in designing a part with mechanical integrity [14]. Therefore, several iterations of a part are typically produced, varying and tuning process parameters for each iteration until the desired part quality is achieved. For a manufacturing technology that targets low-volume or even lot-size productions, finding process parameters experimentally increases the time and cost significantly and is, therefore, undesirable. For example, a survey conducted in 2021 by on-demand manufacturer Hubs shows that 64% of AM production runs only consist of one to ten identical parts [15]. Therefore, having to find process parameters experimentally through iterations can easily multiply the number of parts having to be produced. At the same time, the same survey notes inadequate part quality and limited expertise as two of the main barriers for companies to implement AM [15], further illustrating the need for an objective method to achieve good part quality reliably. Ultimately, such a method could speed up certification processes for AM products, which are currently facing difficulties based on the low repeatability of current AM processes [16].

To overcome the described challenges in setting the correct process parameters, more data has to be acquired on all relevant parameters and their effect on the resulting part’s properties. By including non-linearities and interdependencies, trial designs become very large. For example, even by limiting the regarded process parameters to the eleven parameters identified by Dey et al. to be most important [7] and including part cooling, which is not mentioned in [7] but has been proven to have a large impact on part quality [12,17], a twelve-dimensional trial plan is necessary. With only three set points for each parameter to be able to detect basic non-linearities, this would result in 3^12^ = 531,441 trial points. With this amount of data, it becomes increasingly difficult for humans to detect and understand all results, correlations, causes and interdependencies that can be derived from test results. Even if conclusions can be drawn, using them to actively set and achieve desired part properties requires additional work. Therefore, in this study, an artificial intelligence approach, specifically an Invertible Neural Network (INN)—a technology which has previously not been used in a manufacturing-related context—is used to analyze and evaluate the influence of a subset of relevant process parameters on the resulting part properties. In comparison to other Machine Learning (ML) based approaches, this also enables the inverse use-case of directly generating process parameters to achieve defined part properties.

## 2. Related Work

Finding and setting appropriate process parameters in AM and in other complex manufacturing technologies is often performed by empirical modeling, varying one or more process parameters in a limited range, and observing the effects on the manufactured parts [17,18,19,20,21,22,23]. However, as stated in Section 1 regarding the specific challenge in parameter generation for FDM, varying all relevant parameters results in an unmanageable quantity of trials. To limit the necessary number of trials, the design of the experimental methods is utilized, i.e., Q-optimal trial design [24] or I-optimal trial design [25]. Other approaches base their trials on a face-centered central composite design [26]. Oftentimes, mathematical functions are fitted based on the acquired results [25], sometimes using algorithms inspired by nature, i.e., a bacterial foraging optimization algorithm [27] or evolutionary algorithms [28]. These approaches typically work well for the exact regarded use-case but cannot adapt easily to changing conditions, such as different machines of the same category or different materials. Even for the same conditions, an added process parameter which has previously not been regarded may disrupt the model since parameters for complex manufacturing technologies generally have interdependencies, potentially rendering the model unusable. Furthermore, most studies regarding process set-up in AM only investigate optimizing one or two quality figures, disregarding the effect on other quality figures and, therefore, not considering the trade-off in quality.

To address these issues, newer approaches by Jagadish et al. [29], Jang et al. [30], and Hsieh [31] try to use machine learning-based techniques to perform parameter optimization in different manufacturing processes. Jagadish et al. report a 95% accuracy when comparing predicted parameters for a green manufacturing process to experimental results [29], while Jang et al. achieved an error of less than 1% between prediction and experimental validation for reducing cutting forces in a milling process [30]. Regarding plastics processing, Hopmann et al. demonstrated an approach for setting parameters in injection moulding using artificial neural networks [32]. The study uses a feed-forward neural network, therefore having to check all possible combinations using a brute force approach to then pick a combination of parameters based on the result closest to the desired outcome. The approach demonstrated in this paper omits the need to check every possible combination by introducing Invertible Neural Networks (INN) to production technology, vastly increasing decision speed. Using an INN for parameter generation in FDM also addresses the specific challenges this manufacturing process poses.

## 3. Materials and Methods

### 3.1. Problem Definition

In the context of manufacturing processes, the quality prediction and process parameter proposal problems can be described as the search for two different mapping functions. The first is related to the forward process and relates process parameters and quality measurements. The second is the inverse process which relates quality measurements to process parameters. 

The quality prediction function $f_{y}$ models the well-understood forward process X→Y from a vector $x\;\epsilon \;{\mathbb{R}}^{P}$ containing P process parameters to a vector $y\;\epsilon \;{\mathbb{R}}^{Q}$ containing Q quality measurements. $f_{y}$ summarizes the physical process of setting up a machine with a set of parameters $x_{i}$, executing the predefined manufacturing operation and obtaining the set of quality measurements $y_{i}$ from the manufactured part.

Similarly, the parameter proposal function $f_{x}$ denotes the reverse mapping Y→X. In this mapping, for a desired set of quality measurements $y_{i}$ a set of manufacturing parameters $x_{i}$ is recommended. $f_{x}$ often denotes an optimization over a forward process simulation or the proposals of an expert, which are used to set up a machine or manufacturing process.

These functions are learned from a set M of n ϵ ℕ measured data points. By following the learning process, which is stated as an optimization problem, the two functions’ parameters $\theta_{x}$ and $\theta_{y}$ that maximize a loss are found. This loss L (ex: r^2^, or negative Mean Squared Error (MSE)) measures how well $f_{y}$ and $f_{x}$ model the historical data given the set of parameters $\theta_{x}$ and $\theta_{y}$. Thus, the overall naïve definition of these problems is the search for $f_{y}$, $f_{x}$, $\theta_{x}$ and $\theta_{y}$, as follows:

(1) $$y=f_{y}\left(x;\theta_{y}\right)$$

(2) $$x=f_{x}\left(y;\theta_{x}\right)$$

(3) $$\theta_{y},\;\theta_{x}=\underset{\theta_{y},\;\theta_{x}}{\max}\left(\overset{n}{\underset{i}{\sum }}L_{y}\left(y_{i},f_{y}\left(x;\theta_{y}\right)\right)+L_{x}\left(x_{i},f_{x}\left(y;\theta_{x}\right)\right)\right)$$

For the dataset $M={\left\{\left(x_{0},y_{0}\right),\;\left(x_{1},y_{1}\right),\;\ldots ,\left(x_{n},y_{n}\right)\right\}}_{n}$ of n ϵ ℕ measured data points.

From a data science point of view, the forward process X→Y is well understood and unambiguous. In contrast, the inverse process Y→X is often ambiguous and ill-defined. One of the reasons for this is the loss of information on the forward process. A set of quality measurements is, more often than not, insufficient to describe the dynamics of the physical process. Another reason is the presence of non-monotonic behaviors in the forward process, resulting in multiple sets of parameters yielding the same quality measurements. The lost or hidden information is thus described as a latent vector $z\;\epsilon \;{\mathbb{R}}^{H}$ containing H variables which are important to fully describe the manufacturing process, and which were not measured in the data. Examples of these variables are heat loss, residual stress and resulting microstructure of the materials in additive manufacturing, as well as other unknown dynamics of the physical system.

In order to correct $f_{x}$, the direct and inverse relationship between the manufacturing parameters and the latent variables must also be considered. This adds the complexity of having to estimate $f_{z}$ which models X→Z. The new latent variables must also be considered in $f_{x}$ in order to model Y,Z→X. It must also be mentioned that the real semantic meaning of the resulting vector space Z may not be understood unless the learning of $f_{z}$ is constrained to enforce specific process knowledge. With the latent variables added to the model, the problem definition becomes the search for $f_{y}$, $f_{x}$, $f_{z}$, $\theta_{x}$, $\theta_{y}$, and $\theta_{z}$, as follows:

(4) $$y=f_{y}\left(x;\theta_{y}\right)\;$$

(5) $$z=f_{z}\left(x;\theta_{z}\right)$$

(6) $$x=f_{x}\left(y,\;z;\theta_{x}\right)$$

(7) $$\theta_{y},\;\theta_{z},\;\theta_{x}=\underset{\theta_{y},\;\theta_{z},\;\theta_{x}}{\max}\left(\overset{n}{\underset{i}{\sum }}L_{y}\left(y_{i},f_{y}\left(x;\theta_{y}\right)\right)+L_{x}\left(x_{i},f_{x}\left(y,\;f_{z}\left(x;\theta_{z}\right);\theta_{x}\right)\right)\right)$$

For the dataset $M={\left\{\left(x_{0},y_{0}\right),\;\left(x_{1},y_{1}\right),\;\ldots ,\left(x_{n},y_{n}\right)\right\}}_{n}$ of n ϵ ℕ measured data points, which do not contain measures of the latent variables z.

### 3.2. Invertible Neural Networks

Invertible Neural Networks (INN) prove to be a viable solution for joint quality prediction and parameter proposal problems. These networks were introduced by Ardizzone et al. in order to cope with ambiguous inverse problems [33]. In their work, Dinh et al. introduce “affine coupling layers”, which are the basic building blocks of the models [34]. These layers define a basic invertible equation which has the same number of inputs and outputs. This contributes to the conservation of the information present in the modeled process.

The usage of these networks requires the redefinition of the initially stated problem. First, the desired output vector of the network will be the concatenation of the quality measurements, the latent variables and a padding $y^{\prime }=\left[z,p_{y},y\right]$. Similarly, the input of the network will be the concatenation of the manufacturing parameters and an extra padding $x^{\prime }=\left[x,p_{y}\right]$. The selected dimension of z will define the expected latent variables to encode any extra information in the output, and the dimensions of $p_{x}$ and $p_{y}$ will allow the network to have larger information encodings between layers. Thus, in order to solve jointly $f_{y}$, $f_{x}$, and $f_{z}$, an invertible neural network is used $f_{inn}$.

(8) $$y^{\prime }=\left[z,p_{y},y\right]=f_{inn}\left(x^{\prime };\theta \right)$$

(9) $$x^{\prime }=\left[x,\;p_{x}\right]=f_{inn}{}^{-1}\left(y^{\prime };\theta \right)$$

**Figure 2:**
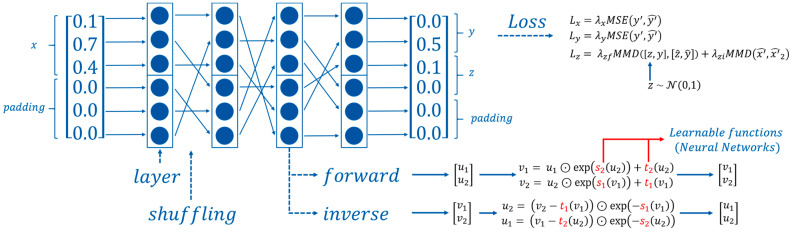
General architecture of the invertible neural network used for process parameter prediction.

In the current work, two joined affine coupling layers are used as the basic layer of the models. In these layers, the input vector is split into two vectors $u_{1}$ and $u_{2}$ which are used to compute the output vectors $v_{1}$ and $v_{2}$. The general architecture of the network is illustrated in Figure 2.

As shown by Ardizzone et al., the base layer function is naturally invertible and allows the forward and inverse learning of the transform functions $t_{1}$,$\;t_{2}$, $s_{1}$, and $s_{2}$ for each layer [33]. These double affine coupling layers are subsequently stacked while shuffling the vectors between layers.

Forward equations:

(10) $$v_{1}=u_{1}⊙\exp\left(s_{2}\left(u_{2}\right)\right)+t_{2}\left(u_{2}\right)$$

(11) $$v_{2}=u_{2}⊙\exp\left(s_{1}\left(u_{1}\right)\right)+t_{1}\left(u_{1}\right)$$

Inverse equations:

(12) $$u_{2}=\left(v_{2}-t_{1}\left(v_{1}\right)\right)⊙\exp\left(-s_{1}\left(v_{1}\right)\right)\;$$

(13) $$u_{1}=\left(v_{1}-t_{2}\left(u_{2}\right)\right)⊙\exp\left(-s_{2}\left(u_{2}\right)\right)$$

The training process of these networks considers performance metrics (losses) for both forward and inverse use of the model, for x, y and z. It must also be noted that the latent variables vector z is not part of the dataset and thus has to be randomly generated for each datapoint from a normal distribution with mean 0 and variance 1, $z_{i}\;\sim \;N\left(0,1\right)$. The padding vectors $p_{x}$ and $p_{y}$ are generated with normal distributions close to zero (ex: mean 0 and variance 1 × 10^−2^).

During the training process, the first step is to use the network to estimate $\hat{y^{\prime }}$. The second step uses the estimated $\hat{y^{\prime }}$ with a small noise (ex: $y_{noise}\;\sim \;N\left(0,\;0.01\right)$) and the inverse of the model to estimate $\hat{x^{\prime }}$. The third step generates a new latent variable $z_{2}$ and replaces it in $\hat{y^{\prime }}$ in order to generate ${\hat{x^{\prime }}}_{2}$.

(14) $$\hat{y^{\prime }}=\left[\hat{z},\hat{p_{y}},\hat{y}\right]=f_{inn}\left(x^{\prime };\theta \right)=f_{inn}\left(\left[x,\;p_{x}\right];\theta \right)$$

(15) $$\hat{x^{\prime }}=\left[\hat{x},\hat{p_{x}}\right]=f_{inn}{}^{-1}\left(\left[\hat{z},\hat{p_{y}},\hat{y}\right];\theta \right)$$

(16) $${\hat{x^{\prime }}}_{2}=\left[{\hat{x}}_{2},{\hat{p_{x}}}_{2}\right]=f_{inn}{}^{-1}\left(\left[z_{2},\hat{p_{y}},\hat{y}\right];\theta \right)$$

These three predictions help compute the three losses of the network $L_{x}$, $L_{y}$, $L_{z}$. The first two supervised MSE losses help approximate X→Y, Z and Y,Z→X. The third unsupervised Maximum Mean Discrepancy (MMD) loss enables a gradual emergence of structure in the latent space Z, and enforces the independence between z and y, so that the same information is not encoded twice [33]. All losses are scaled after the first epoch and the importance of the inverse unsupervised loss ($\lambda_{zi}$) is decreased through training.

(17) $$L_{x}=\lambda_{x}MSE\left(y^{\prime },\;\hat{y^{\prime }}\right)$$

(18) $$L_{y}=\lambda_{y}MSE\left(y^{\prime },\hat{y^{\prime }}\right)$$

(19) $$L_{z}=\lambda_{zf}MMD\left(\left[z,\;y\right],\;\left[\hat{z},\;\hat{y}\right]\right)+\lambda_{zi}MMD\left(\hat{x^{\prime }},\;{\hat{x^{\prime }}}_{2}\right)$$

Thus, the training of the invertible neural network for the joint quality prediction and parameter proposal problems can be denoted as:

(20) $$\theta_{t}=\underset{\Theta }{\max}\left(\overset{n}{\underset{i}{\sum }}\lambda_{x}MSE\left(y^{\prime },\;\hat{y^{\prime }}\right)+\lambda_{y}MSE\left(y^{\prime },\hat{y^{\prime }}\right)+\lambda_{zf}MMD\left(\left[z,\;y\right],\;\left[\hat{z},\;\hat{y}\right]\right)+\lambda_{zi}MMD\left(\hat{x^{\prime }},\;{\hat{x^{\prime }}}_{2}\right)\right)$$

### 3.3. Synthetic Data

Finding correlations via an artificial neural network requires data for training. In combination with the long production times in AM as compared to other production technologies, a complete classification of the process takes a lot of time. To validate the presented approach before conducting all required trials, a synthetic data set based on previously determined correlations can be used. In this study, values for build orientation, infill pattern, infill density, manufacturing speed, nozzle temperature and layer height are regarded as input parameters. They are correlated to the output measurements of part strength, dimensional error, surface roughness and manufacturing time. The correlations are based on the findings of Alafaghani et al. [5], Thrimurthulu et al. [35], Akande [36] and Walsh [6] and are summarized in Table 1.

This way, only linear correlations are regarded, which does not necessarily reflect the actual relation between process parameters and measured part properties. Furthermore, since the correlations are compiled from multiple publications, no interdependencies between process parameters can be detected. Because of this compilation, a mixture of experiments and procedures is regarded, which means that the results are not necessarily comparable and transferable between studies. Therefore, this table of correlations shall be regarded as an artificial case combining relevant results from multiple studies. It cannot be used as a complete classification of the process. It can, however, be used to validate the approach of generating a suitable set of process parameters via an invertible neural network. To increase the accuracy of the predicted process parameters, a complete classification considering non-linearities and parameter interdependencies is still necessary.

### 3.4. Data Acquisition

**Figure 3:**
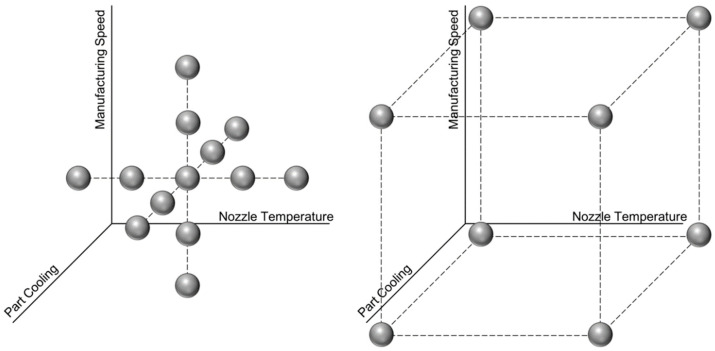
Design of experiments for creating training data. For better illustration, corner points are separated from the star pattern. Combined, the two graphs represent the entire trial plan.

To apply the described procedure to a real manufacturing scenario, the process has to be characterized. For a manageable scope of trials, four FDM process parameters are chosen, which are known to have interdependencies regarding part quality, and which affect mechanical and optical part properties as well as manufacturing time. The chosen parameters are nozzle temperature ($T_{N}$), manufacturing speed ($v_{M}$), part cooling ($C_{P}$) and build orientation ($O_{B}$). For each parameter, a typical range is chosen, which is divided into five equally spaced test points. This means that for nozzle temperature, the distinct values 190 °C, 200 °C, 210 °C, 220 °C and 230 °C are evaluated. Manufacturing speed is varied between 20 mm/s, 35 mm/s, 50 mm/s, 65 mm/s and 80 mm/s, while part cooling is set to 0%, 25%, 50%, 75% and 100%. To account for non-linearities and interdependencies, those three parameters are varied around the centre point of $T_{N}=$ 210 °C, $v_{M}=$ 50 mm/s and $C_{P}=$ 50%, creating a three-dimensional trial set-up in the shape of a star pattern (see Figure 3, left). Additionally, the extreme points in the corners were tested (see Figure 3, right). In combination, this represents a central composite design.

Since build orientation is typically not a parameter with a continuous range but rather depends on the part’s shape, the distinct orientations *lying* (specimen in the x-y-plane) and *standing* (specimen in the x-z-plane) were manufactured for each trial point. Intermediate orientations are technically possible but would require support material to be manufactured. Since the usage of support material increases manufacturing time and needed material significantly while reducing surface quality, its usage should generally be avoided. Therefore, intermediate orientations are not regarded in these trials. All other process parameters were kept at a fixed value throughout the study, according to Table 2. This design of experiments—varying four process parameters with known interdependencies with all other parameters kept constant—allows the validation of the proposed method in a manageable time frame while allowing for easy extension of the learned model when more tests are carried out, i.e., more parameters have been varied in a larger study.

All parts for evaluation are produced on an Ender 3 FDM-3D-printer manufactured by Shenzhen Creality 3D Technology Co., Ltd., Shenzhen, China, using natural Polylactic Acid (PLA) from manufacturer Fillamentum Manufacturing Czech s.r.o., Hulín, Czech Republic. PLA is chosen since it is one of the most used polymers in AM [37], allowing for broad usability of the generated results. In total, five samples are manufactured for each process point to avoid pollution of the results by outliers. To avoid influences of very short layer times, the five samples are printed simultaneously on the same build plate. G-code is prepared using the slicing software Simplify3D in version 4.1.2. For each set of parts, the manufacturing time, as well as the supposed weight of the parts as calculated by the path planning software, is noted, and later compared to the measured values. Furthermore, ambient temperature and humidity are recorded for each test point.

**Figure 4:**
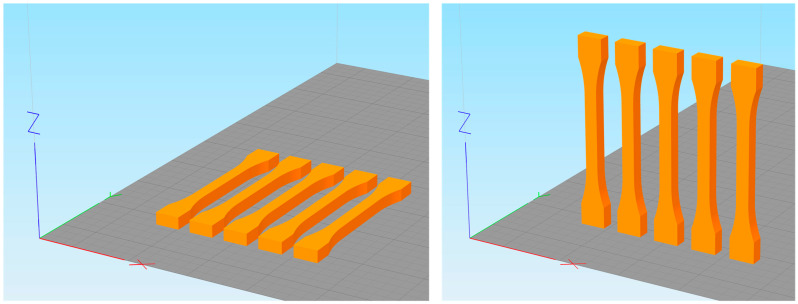
Five tensile test specimens according to DIN EN ISO 527 in lying (**left**) and standing orientation (**right**).

The test geometry to be produced is a tensile test specimen chosen according to DIN EN ISO 527. From this standard, the 1BA test specimen is chosen since it represents a typical test geometry for quantifying the properties of plastic parts and can be produced relatively quickly because of its size. The standard defines the testing zone’s width as 5 mm and its thickness as ≥2 mm. For this study, a thickness of 5 mm was chosen, matching the width of the parts. The test geometry and the manufacturing set-up are displayed in Figure 4.

The tensile test allows for determining tensile strength and Young’s modulus—which are important values to determine part strength—among other characteristics. Before testing the parts using a Z100 tensile testing machine by manufacturer ZwickRoell GmbH & Co.KG, Ulm, Germany, their width and thickness are measured at three points of the testing zone and averaged. Additionally, part weight is measured. For those three values, the deviation from their respective target values is calculated. By defining dimensional accuracy $A_{Dim}$ and weight accuracy $A_{Weight}$ according to Equations (21) and (22), respectively, measured values can be converted into quality indicators for comparing parts and training the INN. To calculate $A_{Dim}$, the measured width $a_{meas}$ and measured thickness $b_{meas}\;$ of the specimen are set in relation to their respective supposed values, $a_{supp}$ and $b_{supp}$. Accordingly, $A_{Weight}$ is calculated from the measured weight of the specimen $w_{meas}$ and its supposed weight $w_{supp}$ as calculated by the path planning software.

(21) $$A_{Dim}=100\%-\frac{\left|a_{supp}-a_{meas}\right|}{a_{supp}}\cdot 100\%-\frac{\left|b_{supp}-b_{meas}\right|}{b_{supp}}\cdot 100\%$$

(22) $$A_{Weight}=\frac{w_{meas}}{w_{supp}}\cdot 100\%$$

This procedure creates the following set (Table 3), which is later used to train the INN. In total, 210 sets of process parameters and corresponding part properties are recorded.

## 4. Validation and Results

To validate the accuracy of the presented INN for process parameter generation, five scenarios are tested, covering different areas of the triangle of goals described in Section 1, Introduction. For each scenario, a set of desired part properties is chosen as input into the previously trained INN. The INN then suggests those process parameters which will most likely produce the desired part properties or part properties close to the desired ones. Furthermore, since it is highly unlikely that the INN finds a set of parameters which fits the desired scenario perfectly and without deviation, a forward calculation is conducted to estimate the resulting part properties based on the suggested process parameters. The parts are manufactured according to the generated parameters and evaluated in the same way as before when creating the training dataset for the INN. Afterwards, the results are compared to the desired input part properties (input accuracy) and to the predicted properties based on the suggested parameters (prediction accuracy). This allows a comparative evaluation of the algorithm. According to Equation (23), input accuracy $A_{Input}$ is defined as the accuracy of the measured values $V_{Measurement}$ compared to the desired values (input) $V_{INN,\;Input}$ the INN is supposed to reach.

(23) $$A_{Input}=100\%-\frac{\left|V_{INN,\;Input}-V_{Measurement}\right|}{V_{Measurement}}\cdot 100\%$$

Prediction accuracy $A_{Prediction}$ is defined according to Equation (24) as the accuracy of the measured values when compared to the predicted values $V_{INN,\;Prediction}$ based on the generated process parameters.

(24) $$A_{Prediction}=100\%-\frac{\left|V_{INN,\;Prediction}-V_{Measurement}\right|}{V_{Measurement}}\cdot 100\%$$

Both accuracy definitions are related to the measured value in the denominator to make them easily comparable.

**Figure 5:**
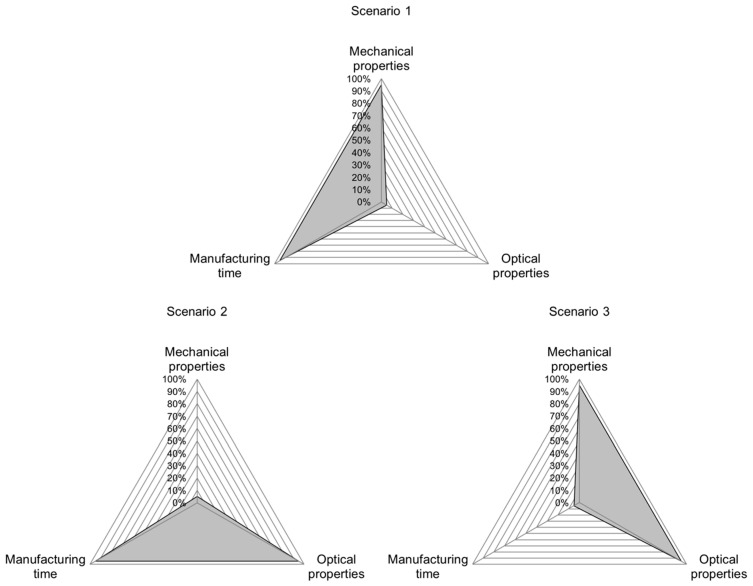
Scenario 1 to 3 used to validate the accuracy of the demonstrated INN. Each scenario favors two distinct part qualities while allowing a compromise in the third. The gray area shows the fulfilment of each quality category.

The validation scenarios are chosen based on the fact that not all part characteristics can be optimized at the same time. For scenario 1, good mechanical properties and a short manufacturing time are demanded, allowing for poor optical properties (Figure 5, top). Figure 5, left, shows the triangle of goals for scenario 2, where good optical properties and a short manufacturing time are demanded while accepting poor mechanical properties. In scenario 3, the demand for good mechanical and optical properties combined, therefore allowing for a long manufacturing time (Figure 5, right).

The exact target values for “good” and “poor” properties are determined based on the range of results acquired in the trials for training the INN. For each part property, the highest and lowest values and the interval in between were determined. “Good” values $V_{good}$ are defined as the upper 5% of the interval between the lowest and highest measured values ($V_{low}$ and $V_{high}$, respectively) of the training set according to Equation (25), while “poor” values $V_{poor}$ are defined as the lower 5% of the interval (Equation (26)). Because manufacturing time is desired to be low, the criteria for good and poor are reversed for this quality characteristic.

(25) $$V_{good}=V_{low}+0.95\cdot \left(V_{high}-V_{low}\right)$$

(26) $$V_{poor}=V_{low}+0.05\cdot \left(V_{high}-V_{low}\right)$$

**Figure 6:**
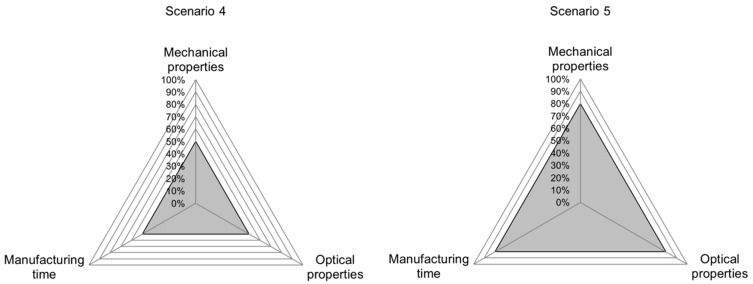
Scenarios 4 and 5 evaluate whether process parameters for medium and above-average part properties can be generated. The gray area shows the fulfilment of each quality category.

In the fourth scenario, it is tested whether medium properties for all regarded part characteristics can be achieved (Figure 6, left). Here, the middle of each interval $V_{medium}$ is used to calculate the desired values (Equation (27)).

(27) $$V_{medium}=V_{low}+0.5\cdot \left(V_{high}-V_{low}\right)$$

Finally, in scenario 5, it is evaluated whether above-average properties can be achieved for all regarded part characteristics (Figure 6, right). Here, $V_{above\;average}\;$ describes the upper 20% of the interval between the lowest and highest value, according to Equation (28).

(28) $$V_{above\;average}=V_{low}+0.8\cdot \left(V_{high}-V_{low}\right)$$

In the regarded use-case of FDM, mechanical properties relate to the part characteristics of Young’s modulus, tensile strength, and elongation at tensile strength, while optical properties relate to the dimensional accuracy as defined in Section 3.4, data acquisition. Since it cannot be indisputably said how weight accuracy influences mechanical and optical properties as well as manufacturing time, weight accuracy is always demanded to be at $V_{medium}$ for each validation trial.

The INN architecture used in these experiments was consistent with the number of inputs and outputs described previously. Moreover, the latent space z was fixed to six dimensions. Including the padding, each complete input and output vector had a total size of 30 which was evaluated through four double affine coupling layers. The transform functions $t_{1}$,$\;t_{2}$, $s_{1}$, and $s_{2}$ for each layer were a set of 512 linear units, then a Relu activation function, and finally 30 linear units. The INN was trained for 100 epochs with batches of 20 shuffled data points. All hyperparameters were selected through a grid search. 

Under these conditions, the target values for the parts being produced according to the parameters generated by the INN are shown in Table 4. For scenarios 2 and 3, target values needed to be adjusted for the INN to generate results. Using the calculated values according to Equations (25) and (26) of 621.67 s and 1522.93 s, respectively, no matching process parameters were found.

To achieve the desired part properties, the INN generated the following sets of process parameters (Table 5, top). A forward calculation is conducted to estimate the resulting part properties when using the generated process parameters. The predicted values are shown in Table 5, bottom. The calculation took less than one second on a regular office laptop, showing its benefit in terms of decision speed.

After manufacturing and evaluating five parts per scenario according to the procedure described in Section 3.4, data acquisition, and obtained results can be compared according to Equations (23) and (24). The results are summarized in Table 6.

Regarding the mean values over all the scenarios, the best input and prediction accuracies can be achieved for part weight with values of 98.38% and 98.45%, respectively. For closely matching the desired input (input accuracy), the category manufacturing time is the least accurate, with a mean value of 71.1%. In terms of prediction accuracy, elongation at tensile strength produces the lowest accuracy at 75.1%.

**Figure 7:**
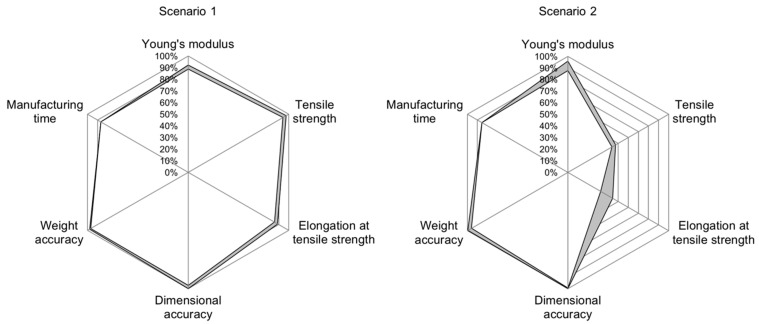
Radar chart for input accuracies in scenarios 1 (**left**) and 2 (**right**). The gray band shows the interval between best and worst accuracy for each quality characteristic.

Analyzing the accuracy values for individual scenarios shows the potential for improving the INN’s accuracy. The INN’s input accuracy is visualized in the radar chart in Figure 5, Figure 6 and Figure 7, allowing a more in-depth analysis of the individual scenarios and the INN’s capability of generating suitable manufacturing parameters to create desired part qualities. The gray band represents the area between the best and worst prediction accuracy for each part characteristic. The same observations can be made for prediction accuracy; however, because input accuracy is more relevant to a real manufacturing use-case, it is focused on in the following evaluation.

Scenario 1 is meant to have good mechanical properties and a low manufacturing time while accepting inferior optical properties. Figure 7, left, shows that the generated process parameters are capable of producing the desired output closely, with the highest accuracy achieved in terms of setting the actual geometry at 99.88%. Even in the worst case of elongation at tensile strength, the INN was capable of matching the desired part characteristics to 85.71%.

In scenario 2, good optical properties and a low manufacturing time are demanded while accepting inferior mechanical properties. Here, the parameters generated by the INN are able to reproduce the categories of Young’s modulus, dimensional accuracy, weight accuracy and manufacturing time closely, as shown in Figure 7, right. However, the generated process parameters do not produce the desired results in the categories of tensile strength and elongation at tensile strength, with accuracy values being as low as 43.67% and 32.63%, respectively.

**Figure 8:**
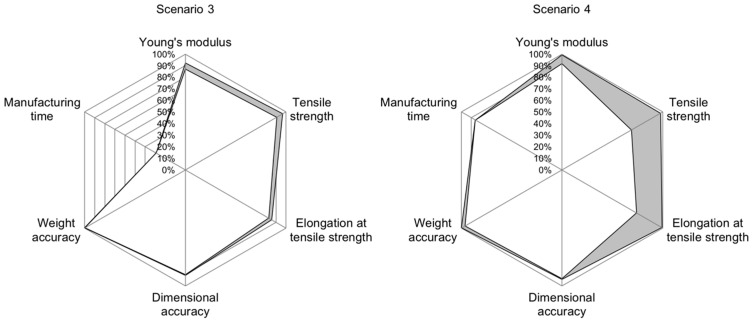
Radar chart for input accuracies in scenario 3 (**left**) and 4 (**right**). The gray band shows the interval between best and worst accuracy for each quality characteristic.

Scenario 3, where manufacturing time was traded for good mechanical and optical properties, generally shows a good accuracy between 82.76% and 99.96%. The only outlier here is manufacturing time, with an accuracy as low as 29.03% (Figure 8, left). 

For scenario 4, where the generation of parameters for creating a part with good medium properties for all categories is demanded, accuracy is good for the categories Young’s modulus, dimensional accuracy, weight accuracy and manufacturing time, as shown in Figure 8, right. The categories tensile strength and elongation at the break still deliver high accuracies of 97.87% and 99.33%, respectively, in the best cases. However, they can be as low as 69.09% and 74.17%, respectively, showing a larger possible range of accuracies for those categories. This can be attributed to a higher standard deviation or, more precisely, one individual outlier in the measured values of this validation trial. 

**Figure 9:**
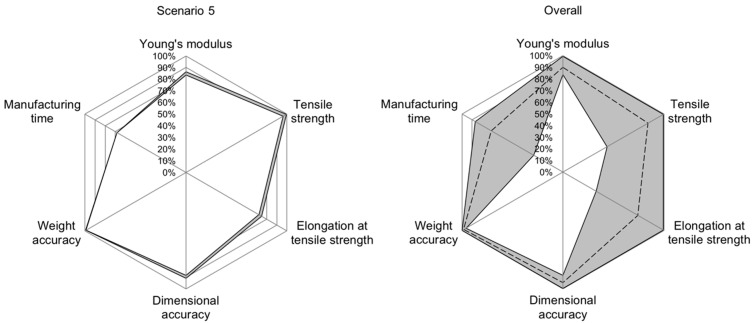
Radar chart for input accuracies in scenario 5 (**left**) and the combination of all scenarios (**right**). The gray band shows the interval between best and worst accuracy for each quality characteristic. The dashed line in the right plot shows the mean value.

For scenario 5, where it is demanded to create an above-average part, input accuracy can be considered good for tensile strength (between 96.08% and 99.49%), dimensional accuracy (between 88.36% and 90.88%) and weight accuracy (between 98.84% and 99.24%). The input accuracy for Young’s modulus is slightly worse at between 83.64% and 86.42%, followed by the input accuracy for elongation at break at between 72.41% and 75% and manufacturing time at 68.26% (Figure 9, left).

Regarding the input accuracy over all scenarios, Figure 9, right, shows that Young’s modulus, dimensional accuracy and weight accuracy can be closely set according to target values when using the presented INN. For categories of tensile strength, elongation at tensile strength and manufacturing time, certain cases still produce a highly accurate result with accuracy values above 99%. However, in the worst cases, accuracies can drop significantly to values as low as 29.03%.

## 5. Summary, Discussion and Outlook

In this study, a method for using invertible neural networks is applied to a manufacturing environment for the first time to automatically generate process parameters based on desired part properties. This is achieved by training an INN using process parameters and corresponding measured part properties. The results of the validation trials show that the presented method is well suited for generating process parameters from a limited dataset to achieve desired part properties. This way, it is possible to objectify decision-making in manufacturing environments while at the same time improving decision quality and decision speed. By objectifying the process set-up for a manufacturing process, constant high-quality production can be maintained because expert knowledge is not bound to individuals. Furthermore, the presented tool can be used to train new employees quickly by showing the effect of process parameters on part quality directly.

In general, the demonstrated INN shows good accuracy in regard to how closely the parts manufactured according to the generated parameters achieve the desired properties, with a mean overall input accuracy of 85.34%. For most cases, it is even higher, reaching up to 99.96% accuracy. The lower mean value is mostly due to the results of scenarios 2 and 3, where inferior mechanical properties and higher manufacturing times were accepted, respectively. Here, the high deviation from otherwise higher accuracy values can be attributed to the artificial construction of the sets of desired part properties. For the cases where one or more attributes were accepted to yield lower results (e.g., set to the lower 5% of the interval) to benefit other factors, those values were deliberately set low, sometimes worse than they would need to be. Given all other constraints, it is difficult to produce parts with such low values for certain categories. This shows the current limitation of the demonstrated INN: all values have to be set, even if certain characteristics take on a subordinate role for the application. As shown in scenarios 2 and 3, this can lead to the overdetermination of the problem when no adequate set of process parameters can be found that accurately reproduces the desired part. Evidence can be found when comparing the desired part quality figures with the INN’s predictions based on its suggested parameters. For example, in scenario 3, where manufacturing time was supposed to be high, the INN struggled to generate a fitting set of process parameters. While the desired manufacturing time was set to 1100 s per part, the INN predicted that it would only achieve a close match of all quality characteristics when producing the part in 985 s. When manufacturing the validation samples, it only took 643.4 s per part for this scenario. This is why, in those cases of overdetermination, prediction accuracy is significantly higher than input accuracy. In a real use-case, it is more likely to define certain important part qualities and leave those that are less relevant unspecified. However, this does not mean that those characteristics are desired to be as poor as possible. They should still be as good as possible under the given restrictions. Therefore, in the next steps, the shown solution needs to be extended by combining INNs with optimization problems. That way, it will be possible to leave out certain part quality characteristics when defining the desired part quality. The INN will then generate a set of process parameters which closely fit the specified quality characteristics while maximizing the unspecified ones. This more closely accounts for a real-world scenario while also solving the current problem of overdetermining the task, ultimately resulting in higher accuracy values. Given those expansions, prediction quality would only depend on the quality of the training data. Therefore, the method could be applied to different polymers, from amorphous to highly crystalline, while prediction quality is expected to stay very high. Additionally, user acceptance and industrial integration of a tool suggesting suitable process parameters for a manufacturing process provide further research perspectives. Based on the presented method’s speed of calculating suitable process parameters given a set of desired part properties, it is possible to run the algorithm on low-power computing hardware, edge devices or directly on the manufacturing machine. This further improves decision speed, decision implementation and automation in manufacturing and shall therefore be investigated in future studies.

## Figures and Tables

**Table 1:** Correlations used to create synthetic data on influence of FDM process parameters. ↑ indicates increase, ↓ indicates decrease.

Input Parameter	Output Measurement
Build orientation is Z	Strength ↓
Infill density ↑	Strength ↑
Manufacturing speed ↑	Strength indifferent
Nozzle temperature ↑	Strength ↑
Layer height ↑	Strength ↑
Infill pattern is hexagonal	Strength ↑
Layer height ↑	Dimensional error ↑
Infill density ↑	Dimensional error indifferent
Manufacturing speed ↑	Dimensional error ↑
Nozzle temperature ↑	Dimensional error ↑
Infill pattern	Dimensional error indifferent
Infill density ↑	Manufacturing time ↑
Infill pattern is linear	Manufacturing time ↓
Infill pattern is hexagonal	Manufacturing time ↑
Manufacturing speed ↑	Manufacturing time ↓
Nozzle temperature ↑	Manufacturing time indifferent
Layer height ↑	Manufacturing time ↓
Infill density ↑	Surface roughness indifferent
Infill pattern	Surface roughness indifferent
Manufacturing speed ↑	Surface roughness ↑
Nozzle temperature very high or very low	Surface roughness ↑
Layer height ↑	Surface roughness ↑

**Table 2:** Process parameters that have not been varied during the study.

Process Parameter	Value
Nozzle Diameter	0.4 mm
Extrusion multiplier	1
Extrusion width	0.48 mm
Retraction	4.9 mm at 55 mm/s
Layer height	0.2 mm
Top solid layers	3
Bottom solid layers	3
Perimeters	2
Perimeter direction	Inside-out
First layer height	150%
First layer width	120%
First layer speed	50%
Start points	X = 0; Y = 500
Skirt	1 Outline (only lying specimen)
Brim	10 Outlines (only standing specimen)
Infill	Rectilinear at 50% infill density; 20% overlap; 120% width; alternating between 45° and −45° each layer
Support	No support
Heated bed	50 °C

**Table 3:** Data used to train the INN.

		Values/Range
**Input that is varied**	Nozzle temperature [°C]	190; 200; 210; 220; 230
	Manufacturing speed [mm/s]	20; 35; 50; 65; 80
	Part cooling [%]	0; 25; 50; 75; 100
	Build orientation [-]	lying, standing
**Input that is not varied**	Every process parameter from Table 2	-
**Input that cannot be influenced**	Ambient temperature [°C]	24.8 to 29.9
	Humidity [%]	34 to 52
**Output**	Tensile strength [MPa]	11.4 to 46.1
	Young’s modulus [MPa]	1872 to 2501
	Elongation at tensile strength [%]	0.52 to 2.5
	Dimensional accuracy [%]	85.2 to 99.8
	Weight accuracy [%]	86.3 to 97.5
	Manufacturing time [s]	571.6 to 1573

**Table 4:** Target values for the produced parts as input into the INN for parameter generation. Adjusted values deviating from the described procedure are marked with *.

Validation Trial	Scenario 1	Scenario 2	Scenario 3	Scenario 4	Scenario 5
Young’s modulus [MPa]	2470.00	1903.00	2470.00	2187.00	2375.00
Tensile strength [MPa]	44.40	13.10	44.40	28.80	39.20
Elongation at tensile strength [%]	2.40	0.62	2.40	1.51	2.10
Dimensional accuracy [%]	85.90	99.10	99.10	92.50	96.90
Weight accuracy [%]	91.90	91.90	91.90	91.90	91.90
Manufacturing time [s]	622.00	1000.00 *	1100.00 *	1072.00	772.00

**Table 5:** Process parameters generated by the INN based on input from Table 4 and predicted part properties when using the generated parameters.

Validation Trial	Scenario 1	Scenario 2	Scenario 3	Scenario 4	Scenario 5
Nozzle temperature [°C]	218	222	202	215	201
Manufacturing speed [mm/s]	36.8	92	47.8	34.1	68
Part cooling [%]	24	100	12	73	19
Build orientation [-]	lying	standing	lying	standing	lying
Young’s modulus [MPa]	2468	1996	2433	2190	2374
Tensile strength [MPa]	44.40	14.80	40.20	29.00	39.10
Elongation at tensile strength [%]	2.64	0.64	2.30	1.51	2.08
Dimensional accuracy [%]	86.10	99.10	93.70	92.70	94.60
Weight accuracy [%]	92.02	91.77	91.80	91.84	91.63
Manufacturing time [s]	622.00	1111.00	985.00	1076.00	771.00

**Table 6:** Results of accuracy analysis, listing input (Equation (23)) and prediction accuracy (Equation (24)) for all scenarios.

		Scenario 1		Scenario 2		Scenario 3		Scenario 4		Scenario 5		Overall			
		min	max	min	max	min	max	min	max	min	max	min	max	Mean	Median
**Young’s** **modulus**	Input accuracy [%]	88.74	92.33	87.70	95.72	86.90	92.23	91.73	99.26	83.64	86.42	83.64	99.26	89.85	90.56
	Prediction accuracy [%]	88.83	92.41	91.98	99.60	88.60	93.85	91.58	99.12	83.68	86.47	83.68	99.60	91.04	91.58
**Tensile strength**	Input accuracy [%]	94.29	97.22	43.67	47.46	90.91	96.74	69.09	97.87	96.08	99.49	43.67	99.49	84.11	94.79
	Prediction accuracy [%]	94.29	97.22	49.33	53.62	93.49	98.77	68.18	97.16	95.83	99.49	49.33	99.49	85.16	94.29
**Elongation/** **at tensile** **strength**	Input accuracy [%]	85.71	88.89	32.63	44.29	82.76	85.71	74.17	99.33	72.41	75.00	32.63	99.33	73.96	82.76
	Prediction accuracy [%]	94.29	97.78	33.68	45.71	79.31	82.14	74.17	99.33	71.72	74.29	33.68	99.33	75.10	79.31
**Dimensional accuracy**	Input accuracy [%]	97.25	99.88	99.29	99.90	90.13	90.86	93.81	94.39	88.36	90.88	88.36	99.90	94.65	94.39
	Prediction accuracy [%]	97.01	99.88	99.29	99.90	96.12	96.81	94.02	94.59	91.01	93.47	91.01	99.90	96.39	96.58
**Weight** **accuracy**	Input accuracy [%]	96.90	97.73	95.96	99.41	99.60	99.96	96.34	99.74	98.84	99.24	95.96	99.96	98.38	98.48
	Prediction accuracy [%]	97.03	97.86	95.82	99.55	99.71	99.98	96.27	99.80	99.14	99.54	95.82	99.98	98.45	98.41
**Manufacturing time**	Input accuracy [%]	86.75	86.75	85.56	85.56	29.03	29.03	85.91	85.91	68.26	68.26	29.03	86.75	71.10	85.56
	Prediction accuracy [%]	86.75	86.75	95.05	95.05	46.91	46.91	86.23	86.23	68.43	68.43	46.91	95.05	76.67	86.23

## Data Availability

The data presented in this study are available on request from the corresponding author.
